# Trends in adherence to the 24‐h movement guidelines among US adolescents from 2011 to 2019: Evidence from repeated cross‐sectional cycles of the Youth Risk Behavior Surveillance System

**DOI:** 10.1111/sms.14609

**Published:** 2024-03-27

**Authors:** Sitong Chen, Denver Brown, Kate Parker, Eun‐Young Lee

**Affiliations:** ^1^ Institute for Health and Sport Victoria University Melbourne Victoria Australia; ^2^ Department of Psychology The University of Texas at San Antonio San Antonio Texas USA; ^3^ School of Exercise and Nutrition Sciences Deakin University Geelong Victoria Australia; ^4^ School of Kinesiology and Health Studies Queen's University Kingston Ontario Canada

**Keywords:** movement behaviors, physical activity, screen time, sleep, time‐use epidemiology, trend analysis

## Abstract

**Background:**

Adherence to the 24‐h movement guidelines is associated with various health benefits, but given the novelty of these integrative recommendations, little is known about year‐to‐year trends in guideline adherence in adolescents. This study investigated trends of adherence to the 24‐h movement guidelines among US adolescents.

**Methods:**

Data from 2011 to 2019 cycles of the Youth Risk Behavior Surveillance System were used, which included 62 589 US adolescents aged 14–17 years (female: unweighted sample size = 31 876, 51%; weighted% = 50.1%). Participants self‐reported their demographic information (i.e., sex, age, race/ethnicity), physical activity, screen time and sleep duration. Meeting the 24‐h movement guidelines was operationalized as simultaneously engaging in 60 min or more of moderate‐to‐vigorous physical activity, no more than 2 h of screen time, and 8–10 h of sleep per day. Trend analysis was used to examine the secular changes in adherence to the integrated guidelines from 2011 to 2019.

**Results:**

Downward trends in adherence to the 24‐h movement guidelines were observed among adolescents from 2011 (3.6%) to 2019 (2.6%). After stratification by sex, age, and race/ethnicity, similar downward trends in the guideline adherence were observed in females and Black/African American adolescents. The lowest prevalence of meeting the individual guidelines was for the PA guidelines (25.6%). Movement guideline adherence was consistently lowest among females, older adolescents, and those who identified as Black/African American.

**Conclusions:**

Adherence to the 24‐h movement guidelines has declined among US adolescents over the past decade. Interventions should prioritize an integrative approach that could increase concurrent adherence to each of the 24‐h movement guideline, particularly among female, older and minority adolescents.

## 1 BACKGROUND

Research integrating physical activity (PA), sedentary behavior (SB), and sleep has been gaining traction globally.^1^, ^2^ From a 24‐h movement behavior perspective, PA, SB and sleep co‐dependently influence health; therefore, favorable compositions of these behaviors (e.g., high PA, low SB, adequate sleep) are most desirable.^3^ For example, recent work has demonstrated that considering all three movement behaviors concurrently can help researchers and health professionals better understand associated health outcomes at the population level.^1^, ^4^

Several governments and organizations have released 24‐h movement guidelines specific to different segments of the population (e.g., children and youth, adults, older adults).^5^, ^6^, ^7^ Specifically, the first 24‐h Movement Guidelines for Children and Youth (5–17 years) (24‐h movement guidelines hereafter) were developed in Canada and released in 2016.^5^ These guidelines recommend that adolescents (14–17 years) should accumulate at least 1 h of daily moderate‐to‐vigorous PA (MVPA), limit recreational screen time (ST) to no more than 2 h per day, and obtain 8–10 h of sleep per night.^5^ Based on these guidelines, an increasing number of studies have investigated the prevalence and correlates of guideline adherence as well as associated health outcomes.^2^, ^8^, ^9^, ^10^, ^11^, ^12^ This emerging body of literature has established an evidence base from which resources can be directed to improve public health through modifying 24‐h movement behavior patterns.

Some systematic reviews have highlighted the importance of adhering to the 24‐h movement guidelines for adolescent health.^2^, ^13^ Given this, continued surveillance and monitoring adherence to the 24‐h movement guidelines is a priority for public health.^2^ Researchers from many countries have reported the prevalence of 24‐h movement guidelines adherence for adolescents.^8^, ^9^, ^10^ A meta‐analysis of 387 437 participants from 23 countries found that only 2.7% (95% Confidence Interval [CI], 1.8–3.6) meet all three recommendations within the guidelines concurrently.^12^ North American data has shown that 5.0% (95% CI, 4.6–5.4) of US adolescents met the all three guidelines,^8^ whereas for Canadian adolescents, the corresponding percentage was 2.6%.^9^ These results suggest that adherence to the guidelines is generally very low for adolescents, which may pose significant public health problems given patterns of movement behaviors could be established in adolescence and track into adulthood.^11^ In order to develop effective public health interventions, there is a research need to understand salient factors associated with adherence to the guidelines. Previous studies have identified that sex,^10^, ^12^, ^14^ age,^10^, ^11^, ^12^ race,^10^, ^15^ parental education,^10^, ^15^ family socioeconomic status^10^, ^15^ are correlated with meeting the guidelines. However, correlates of 24‐h movement guideline adherence have received limited attention in US adolescents.

Although many previous studies have used nationally representative samples to estimate the prevalence of 24‐h movement guideline adherence among adolescents,^10^, ^15^, ^16^ for the most part, these studies have used cross‐sectional data (survey at one time point). Thus, a major knowledge gap exists regarding secular trends due to a limited evidence base of how patterns of 24‐h movement behaviors track over time. Using a repeated cross‐sectional design allows researchers to track population‐level changes in adherence to public health recommendations over time without being affected by attrition as typically observed in cohort studies, which can reduce generalizability of findings.^17^ Repeated cross‐sectional designs can also be more cost‐effective and less resource‐intensive than studies that involve tracking individuals over multiple data collection waves.^18^ For these reasons, repeated cross‐sectional design studies are viewed as one of the strongest ways to capture rates of public health guideline adherence such as rates of youth meeting the 24‐h movement guidelines. While previous research has documented trends in adherence to MVPA,^19^, ^20^ ST^21^, ^22^ and sleep^23^ guidelines in isolation based on past independent public health recommendations, the novelty of the 24‐h movement paradigm warrants greater research attention while taking an integrated approach.^1^

Our current understanding of secular trends in healthy movement behaviors remains limited. This is problematic considering we live in a fast‐paced, fast‐changing society where population level behavioral patterns may change rapidly. Moreover, because of the considerable positive health implications of meeting the 24‐h movement guidelines,^2^ it is important to comprehensively understand these trends during adolescence, which represents an important life stage when long‐term behavioral patterns and health are being established.^24^

Evidence also shows that secular changes of healthy behaviors vary across sub‐population groups (e.g., by sex), such as PA.^25^ Therefore, a similar pattern may be observed when examining the secular trends in meeting the 24‐h movement guidelines; but little is known about this research question in the current literature. Thus, the purpose of this study was to examine trends in adherence to the 24‐h movement guidelines in comparable nationally representative samples of US adolescents over time and by sociodemographic characteristics. A secondary study aim was to examine demographic correlates of 24‐h movement guidelines adherence. Findings of this study could provide policymakers with key information that can be used to implement effective strategies for promoting 24‐h movement behaviors and tailoring health promotion strategies towards subgroups within the population who may be of most need.

## 2 METHODS

### 2.1 Study design and population

This study used data from five cycles of the Youth Risk Behavior Surveillance System (YRBSS; 2011, 2013, 2015, 2017, 2019). The YRBSS is a biennial, cross‐sectional school‐based survey of health‐related behaviors among a nationally representative sample of high school students living in the US.^26^ The YRBSS uses a three‐stage cluster sampling design to recruit students attending public and private schools in grades 9–12 (age range: 12 years or younger, 13, 14, 15, 16, 17, and 18 years or older).^26^ The survey was administered in person by trained data collectors and completed by students during school hours.

The present study included YRBSS cycles dating back to 2011 as this cycle represented the first administration in which MVPA, recreational ST, and sleep duration were measured concurrently. The initial sample consisted of 73 074 participants. Among them, participants aged 12 or below (0.3%), 13 years (0.1%), and 18 years or older (13.9%) were excluded. A total of 62 589 participants aged 14–17 years were included for the final analysis. The overall response rate was above 60% during the administration of each cycle of the YRBSS. Survey results were weighted to be nationally representative. The YRBSS survey was approved by the Institutional Review Board of the Centre for Disease Control (CDC), US. Written informed consent to participate in this study was provided by the participants' legal guardian/next of kin. Additional details about the YRBSS can be found by accessing the study protocol.^26^

### 2.2 Measures

#### 2.2.1 Demographics

Participants provided demographic information pertaining to their sex, age and race/ethnicity, and their information was classified into sex (female and male), age (14‐, 15‐, 16‐ and 17‐year‐old) and race/ethnicity (White, Black/African American, Hispanic/Latino, and all other races).

#### 2.2.2 Movement behaviors

Movement behaviors were operationalized based on the Canadian 24‐h Movement Guidelines for Children and Youth.^3^ Our analyses focused on the three threshold‐based movement behavior guidelines: MVPA, recreational ST, and sleep. Items used to assess each of these movement behaviors have shown acceptable reliability and validity in previous epidemiological studies.^27^

#### 2.2.3 MVPA

Participants responded to one item that asked: “During the past seven days, on how many days were you physically active for a total of at least 60 min per day.” Response options included “0, 1, 2, 3…7 days.” Responses were dichotomized to represent whether participants met the MVPA recommendation of engaging in ≥1 h of MVPA per day or not.^5^

#### 2.2.4 Recreational ST

Participants responded to two items that asked: “On an average school day, how many hours do you (1) watch TV, and (2) play video or computer games or use a computer for something that is not schoolwork? (Included activities such as Nintendo, Game Boy, PlayStation, Xbox, computer games, and the Internet).” Response options included “I do not watch TV/play video or computer games or use a computer for something that is not schoolwork on an average school day”, “Less than 1 h per day,” “1 h per day,” “2 h per day,” “3 h per day,” “4 h per day” and “5 or more hours per day,” For both items, “less than 1 h per day” was set as 0.5 h and “5 or more hours per day” as 5 h. This allowed us to sum the ST hours to create a single item representing the total amount of recreational ST. Responses were dichotomized to represent whether participants met the recreational ST recommendation of engaging in ≤2 h of ST per day or not.^5^

#### 2.2.5 Sleep

Participants responded to one item that asked: “On an average school night, how many hours of sleep do you get?” Response options included “4 or less hours.” “5 h”, “6 h”, “7 h”, “8 h”, “9 h” or “10 or more hour.” Although sleep recommendations suggest 8–10 h for adolescents between the ages of 14–17 years old, the nature of the greatest response option for sleep (i.e., 10 or more hours) forced an analytical decision that is not in perfect alignment with the guidelines. Responses were therefore dichotomized to represent whether participants met the sleep recommendation if they reported eight or more hours of sleep per night, or not.^5^

Based on the criteria of meeting the PA, ST and sleep guidelines, participants were classified as meeting the 24‐h movement guidelines if they reported meeting the PA, recreational ST and sleep guidelines concurrently^5^; on the contrary, participants were classified as not meeting the 24‐h movement guidelines if they reported not meeting any or all of the three individual components.^5^

### 2.3 Statistical analysis

All the variables included in this study were treated as categorical. Missing values for the study variables of interest ranged from 0.6% (for age) to 15.2% (for adherence to the 24‐h movement guidelines). To avoid biases due to missing data, we implemented multiple imputations by chained equations.^28^ We selected 20 imputations on the basis of the general rule that the number should be at least as large as the percentage of missing data.^28^ The imputed descriptive statistic values closely matched the original observed values without significant differences in all the studied variables. For each variable, then, weighted prevalence estimates with 95% CIs were calculated while taking into account complex sampling survey, by using Taylor linearization to produce nationally representative prevalence estimates for each survey year. To examine trends in the prevalence of meeting the 24‐h movement guidelines, and prevalence of meeting the PA, ST and sleep guidelines separately, across the 2011–2019 cycles of the YRBSS, logistic regression models were conducted with time‐trend variables that assess linear and quadratic (U‐shaped) changes across the five cycles of data collection. Separate logistic regression models were also performed to explore the associations between demographic variables (sex, age, and race/ethnicity) and adherence to the 24‐h movement guidelines and PA, ST and sleep guidelines separately, which generated year‐based and year‐combined associations. Adjusted odds ratio (OR) with 95% CIs, controlling for sex, and race/ethnicity, were presented for all logistic regression models, depending upon which subgroup was being analyzed. All analyses were performed using SVY procedures in Stata/IC 18.0 BE (Stata Corp LLC). Statistical significance was based on a 2‐tailed *p*‐value of less than 0.05.

## 3 RESULTS

**FIGURE 1:**
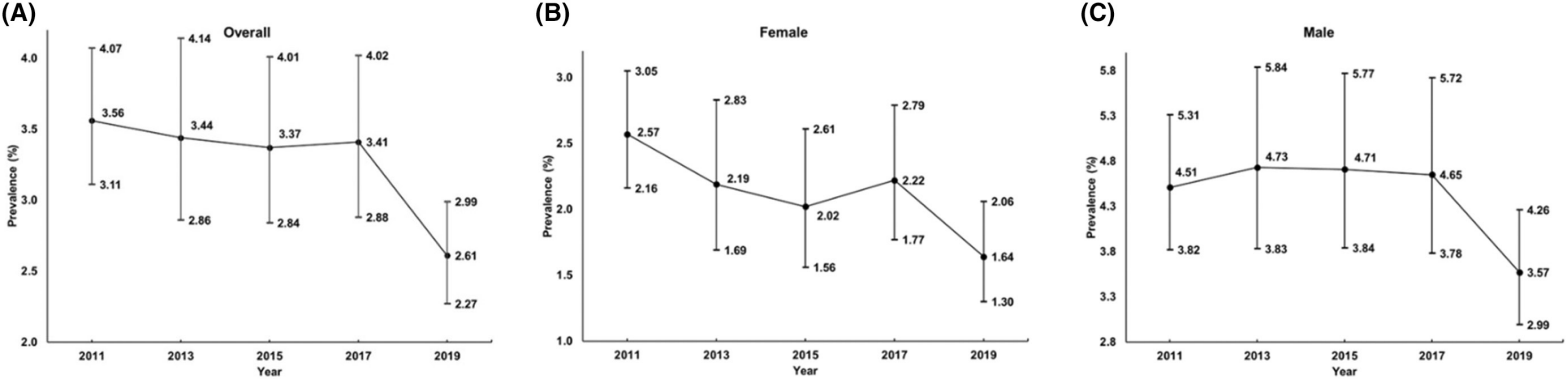
Concurrent prevalence of the adherence to all three threshold‐based 24‐h Movement Guidelines in the overall sample and stratified by sex. (A) In the overall sample, (B) In female sample, (C) In male sample. The analysis for the overall sample was adjusted for sex, age and race/ethnicity. The sex‐split analyses were adjusted for age and race/ethnicity.

**FIGURE 2:**
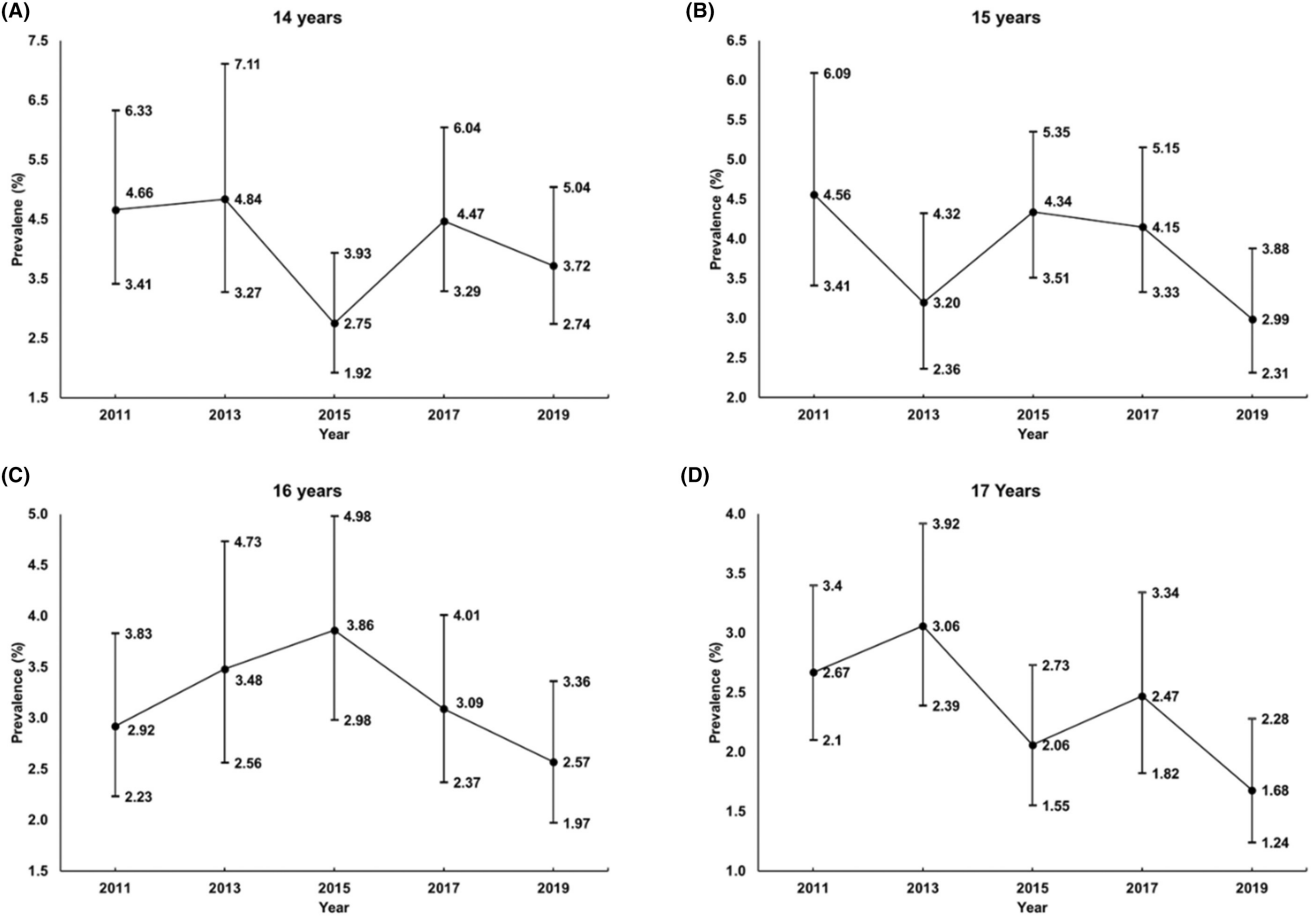
Concurrent prevalence of the adherence to all three threshold‐based 24‐h Movement Guidelines by age group. (A) In the sample of 14 years, (B) In the sample of 15 years, (C) In the sample of 16 years, (D) In the sample of 17 years. The age‐split analyses were adjusted for sex and race/ethnicity.

**FIGURE 3:**
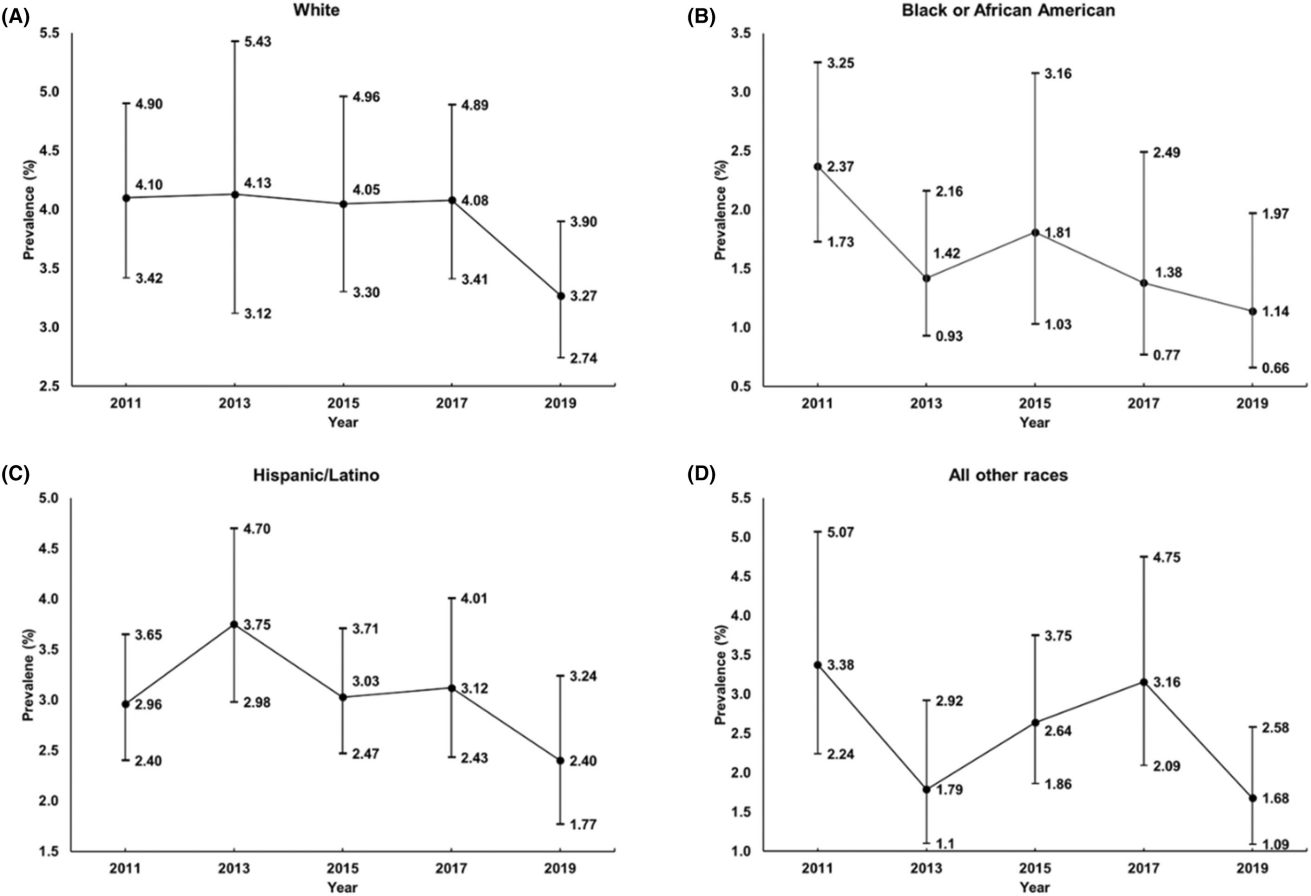
Concurrent prevalence of the adherence to all three threshold‐based 24‐h Movement Guidelines by race/ethnicity, (A) In the sample of White, (B) In the sample of Black/African American, (C) In the sample of Hispanic/Latino, (D) In the sample of all other races. The race/ethnicity‐split analyses were adjusted for sex and age. All other races included American Indian/Alaska Native, Asian, Native Hawaiian/other Pacific Islanders, and Multiple—Non‐Hispanic.

Sample characteristics for the overall pooled sample as well as each sample stratified by their respective YRBSS cycle are presented in Table 1. Overall, 62 589 participants were included in this study, of whom females accounted for 50.1% (weighted result, 95% CI, 49.1–51.1) and the majority of participants identified as White (53.9%, 95% CI, 51.3–56.6). The proportion of 14‐, 15‐, 16‐ and 17‐years participants was 12.9% (95% CI, 12.2–13.6), 29.0% (95% CI, 28.4–29.6), 30.2% (95% CI, 29.6–30.8) and 27.3% (95% CI, 27.3–28.5). The prevalence of meeting the PA, ST and sleep guidelines was 26.9%, 27.7%, and 28.5%, respectively (weighted results). The overall prevalence of meeting the 24‐h movement guidelines was 3.3% (weighted result, 95% CI, 3.1–3.5). More information on samples of different cycles can be found in Table S2. Trends for 24‐h movement guideline adherence in the overall sample and sample stratified by sex, age, and race/ethnicity are illustrated in Figures 1, 2, 3. Figure 1 demonstrates a generally declining (OR: 0.95, 95% CI, 0.90–0.99, *p*_for linear trend_ = 0.016) but slightly fluctuating trend in the overall sample and similar declining patterns were also observed in females (OR: 0.92, 95% CI, 0.86–0.98, *p*_for linear trend_ = 0.012). Figure 2 shows the trends for adherence to the 24‐h movement guidelines stratified by age. The results showed fluctuating but generally downward trends between 2011 and 2019 in all age groups. A quadratic trend was only observed in the 16‐year‐old sub‐group (OR: 0.92, 95% CI, 0.85–0.99, *p*_for quadratic trend_ = 0.030). Figure 3 shows the trends for adherence to the 24‐h movement guidelines stratified by race/ethnicity. Results demonstrated significant negative linear trends for adolescents who identified as Black/African American (OR: 0.85, 95% CI, 0.73–0.99, *p*_for linear trend_ = 0.034); for other races/ethnicities, differently fluctuating (Hispanic/Latino and Other) and relatively stable (White) trends were observed.

**TABLE 1:** Demographic characteristics of study participants.

	Total		Weighted		
	*n*	%	%	95% CI	
Total	62 589	100.0	/	/	
Sex					
Female	31 890	51.0	50.1	49.1	51.1
Male	30 699	49.0	49.9	48.9	50.9
Age group					
14 years	8234	13.2	12.9	12.2	13.6
15 years	17 444	27.9	29.0	28.4	29.6
16 years	18 971	30.3	30.2	29.6	30.8
17 years	17 940	28.7	27.9	27.3	28.5
Race/ethnicity					
White	27 833	44.5	53.9	51.3	56.6
Black or African American	10 558	16.9	13.6	12.3	15
Hispanic/Latino	17 224	27.5	22.7	20.7	24.7
All other races^a^	6974	11.1	9.8	8.8	10.8
Physical activity guidelines					
Not met	45 771	74.4	73.1	72.3	74.0
Met	16 818	25.6	26.9	26.0	27.7
Screen time guidelines					
Not met	44 681	72.0	71.4	70.4	72.3
Met	17 908	28.0	28.6	27.7	29.6
Sleep guidelines					
Not met	44 760	71.7	71.5	70.6	72.4
Met	17 829	28.3	28.5	27.6	29.4
24‐h movement guidelines					
Not met	60 640	96.9	96.7	96.5	96.9
Met	1949	3.1	3.3	3.1	3.5

Abbreviation: CI, confidence interval.

^a^ All other races included American Indian/Alaska Native, Asian, Native Hawaiian/other Pacific Islanders, and Multiple—Non‐Hispanic.

In Table S3, results for the associations between demographic characteristics and meeting the 24‐h movement guidelines in the combined sample and sample by different survey cycles are shown. In the combined sample, compared with females, male adolescents were more than twice as likely to meet the guidelines (OR: 2.16, 95% CI, 1.89–2.46). Also, younger adolescents were more likely to meet the guidelines when comparing with their oldest counterparts (17 years). Specifically, those aged 14 years had the highest likelihood of meeting the guidelines (OR: 1.85, 95% CI, 1.51–2.26). Statistically significant differences for adherence to the guidelines across different races/ethnicities were also found. Adolescents who identified as White, Hispanic/Latino and All other races/ethnicities tended to report meeting the guidelines at higher rates compared to those who identified as Black/African American. The results by each cycle can also be found in Table S3. Table S4 shows the results for trend analysis of each movement behavior separately.

## 4 DISCUSSION

The current study investigated trends in the prevalence of 24‐h movement guideline adherence among samples of US adolescents using five cycles of data from the YRBSS (2011 to 2019). Adherence to the 24‐h movement guidelines was low within each of the survey cycles, with trend analysis revealing a downward trend in adherence to the guidelines over the eight‐year period of investigation (3.6% in 2011–2.6% in 2019). Adherence rates to the three individual guidelines (i.e., PA, ST, and sleep) was low, with the lowest prevalence for the PA guidelines. Changes in adherence to the three individual guidelines by different subgroups were nearly presented as a linear trend (declining trend for PA and sleep guidelines while increasing trend for ST guidelines). The prevalence of guideline adherence was higher for males than females, however, similar declining trends were observed in only the female group. Although guideline adherence was lower with increasing age, age‐stratified analyses showed a general decline, but with differentiated fluctuations, in the 24‐h movement guideline adherence among US adolescents. Differences in trends were also observed for race/ethnicity with those who identified as Black/African American showing a downward trend over time while their White counterparts showed the most stable trend patterns of the guideline adherence over time.

Regardless of survey year, our results found concurrent adherence to each of the 24‐h movement guidelines is low among US adolescents. These findings align with the results from a recent systematic review and meta‐analysis based on international data.^12^ Furthermore, our results are comparable with prevalence estimates of 24‐h movement adherence reported in nationally representative population level studies from other countries. For example, a cross‐sectional study based on Chinese children and adolescents found that only 5.2% met all three threshold‐based movement guidelines.^10^ However, the prevalence was lower than the results based on the Canadian Health Measure Survey^29^, ^30^ but slighter higher than the level during the COVID‐19 according to the 2022 Report Card data.^31^ This may be owing to different measurements and survey time points. For example, adolescent had fewer structured and unstructured opportunities to participate in PA during the pandemic, which further resulted in a lower level of the prevalence of meeting the movement guidelines. Despite variations in adherence to the guidelines across different studies, which may be owing to the use of different measures and data collection windows, collectively, evidence indicates adherence is low and needs to be enhanced for health promotion. It is worth noting that in the combined sample, adherence rate to the three individual guidelines (i.e., PA, ST, and sleep), the lowest one was PA guideline adherence (25.6%), which might be the strongest driver of the low adherence rate of the 24‐h movement guidelines. This finding underscores the central role of PA when promoting healthy 24‐h movement behaviors in adolescents. However, when examining some specific survey years, the individual guidelines with the lowest prevalence varied. This finding should be considered when analyzing the main driver of low 24‐h movement guideline adherence. It has to be acknowledged that the definition of meeting the PA guidelines in this study (i.e., at least 60 min a day on each day of the week)^3^ was different from the current World Health Organization PA guidelines (i.e., weekly average of 60 min a day) for children and adolescents.^32^ This may be an explanation for the relatively lower prevalence of meeting the PA guidelines in this study compared to population‐based studies of youth from other countries. Even so, findings of this study present a significant health threat, as ample evidence has demonstrated the strong association between 24‐h movement guideline adherence and various health outcomes.^2^ Our study provides an impetus for the development of effective health promotion strategies that take an integrative approach targeting MVPA, recreational ST and sleep simultaneously, as there is evidence suggesting a declining trend in rates of 24‐h movement guideline adherence.^33^

The present study provides initial evidence that adherence to the 24‐h movement guidelines has declined among US adolescents over the past decade. Other nations are experiencing similar public health concerns related to declining movement behavior guideline adherence as evidenced by a recent study of Australian adolescents.^33^ Together, these findings extend upon previous work that has investigated MVPA, recreational ST and sleep trends independently, each showcasing less favorable outcomes.^34^, ^35^, ^36^ Taking an integrative approach rooted in the 24‐h movement paradigm represents a strength of the present study as more evidence emerges demonstrating the need to consider the co‐dependence among these behaviors and how they interact to influence health.^2^ Reversing the downward trends is urgently required to improve health among US adolescents.

Our study indicates that the PA guidelines and sleep guidelines adherence in US adolescents has declined over the past years. These findings are consistent with the previous studies,^19^, ^20^, ^23^, ^36^ highlighting that sufficient PA and adequate sleep duration should be promoted for health consideration during adolescence. However, ST guidelines adherence generally displayed as an increasing trend, which is inconsistent with some countries' data.^20^, ^22^, ^33^, ^34^ This finding should be interpreted with caution, given the ST measures in this study only consider TV and computer use while did not consider smartphone and tablet use that have been a popular ST exposure source of adolescents.^22^ Considering the increasing trend in adherence to the ST guidelines in this study, on the basis of the increasing trend in adherence to the ST guidelines, to reverse the declining trends in adherence to the 24‐h movement guidelines, PA and sleep guideline adherence rates with time should be priority. Further, when looking at the changes of PA and sleep guideline adherence rates between 2011 and 2019, we found that the declining change in the sleep guideline adherence rate was larger than that in PA guidelines adherence regardless of subgroups. The analysis on why the difference in sleep guideline adherence was larger than that for the PA guideline would be beyond the current study and this finding might be useful in demonstrating the potentially strongest driver that contributes to the declining trend of 24‐h movement guidelines adherence. Taken together, based on the above analysis, it is important to consider improving PA and sleep duration of adolescents, especially sleep, in order to optimize healthy 24‐h movement behaviors.

The subgroup analyses conducted in this study provide some noteworthy insights into where potential intervention efforts may have the greatest impact based on demographic characteristics. Sex‐based differences in 24‐h movement guideline adherence were demonstrable across the interval of observation. Specifically, and despite very low adherence rates among both sexes, males had over double the prevalence of adherence to the 24‐h movement guidelines compared to females. Evidently, sex is an important correlate of movement behaviors patterns among US adolescents. This finding was consistent with previous studies,^10^, ^37^ which highlights the need to prioritize promoting healthy movement behavior patterns among females during adolescence.^38^

Differences across race/ethnicity were also observed. According to our results, adherence to the 24‐h movement guidelines was highest for adolescents who identified as White, followed by those who identified as Hispanic or Other, and lastly, those who identified as Black/African American. These findings were mostly constant across survey cycles. This finding is consistent with a previous study, suggesting that White adolescents have greater adherence to the 24‐h movement guidelines compared to their non‐White counterparts.^10^ Likewise, previous research has reported that White adolescents are more likely to engage in healthy patterns of movement behaviors when studied in isolation (e.g., sufficient levels of PA^39^). Based on these results, we could imply that White adolescents may exhibit better health outcomes. Race/ethnicity‐related health disparities in adolescents are a concerning public health problem in many countries.^40^ Based on the intersectionality framework,^41^ in racially/ethnically diverse countries, race/ethnicity is often times intertwined with other identities such as gender, income, class or immigrant status that make certain population groups more vulnerable in terms of access to resources and services such as knowledge that people can engage in healthy movement behaviors.

Corroborated by meta‐analytic evidence,^12^ age was also found to be an important demographic correlate associated with 24‐h movement guideline adherence. The lowest rates of adherence to the 24‐h movement guidelines, across all timepoints, were observed among the oldest adolescents (17 years). There are several potential reasons why older adolescents meet the 24‐h movement guidelines at lower rates than their younger peers. One reason may be that older adolescents take on more competing priorities (e.g., academic work, dating, and employment) and therefore cannot dedicate enough time to being active that is required to meet the PA guidelines.^42^ Of note, the reasons for the age‐related decline in trends of adherence to the 24‐h movement guidelines among US adolescents may be different from that in other countries and/or populations owing to social and cultural differences. More studies are, therefore, encouraged to explore the reasons why older US adolescents had lower adherence to the 24‐h movement guidelines. These findings would be beneficial to develop strategies to promote healthful movement behaviors in adolescents as they get older to support more favorable behavioral transitions into adulthood.

## 5 STUDY STRENGTHS AND LIMITATIONS

Some study strengths are worthwhile to mention. First, our study is the first to demonstrate secular trends for adherence to the 24‐h movement guidelines among US adolescents based on the most up‐to‐date nation‐wide surveys. Second, trend analyses were also computed based on demographic parameters (i.e., sex, age, race/ethnicity), which can extend our understanding of healthy movement behaviors for the population health perspective. Third, this study was conducted using a nationally representative sample of US adolescents, and therefore, our findings can demonstrate higher generalizability.

Some limitations inherent to the study data should also be acknowledged. As the YRBSS is a national surveillance study, a self‐reported questionnaire is the most feasible measure, which is subject to respondents' recall bias and social desirability. Additionally, our total sample consisted of different participants measured in each survey cycle, which resulted in multiple cross‐sectional estimates. Future research using data from longitudinal cohorts in diverse populations is warranted to understand how patterns of movement behavior guideline adherence develop through adolescence and would allow for the uncoupling of between‐ and within‐participant effects. Moreover, it is currently recommended that adolescents should accumulate MVPA with an average of 60 min per day,^32^ but given the precluded data it is impossible to estimate the adherence rate to the new MVPA recommendation. This is a limitation inherent to the current study. Although our study sought to reduce estimation bias through including sex, age and race/ethnicity as covariates, several variables known to be associated with movement behaviors were not included (e.g., gender, household income, parental education). To reduce bias in future estimates, studies should prioritize including other sociodemographic as well as environmental variables, such as household income and neighborhood characteristics, that may confound estimates for 24‐h movement guideline adherence. Another limitation was that the YRBSS sleep item only focused on sleep duration on school nights, which fails to consider the variation in sleep duration that occurs on weekends. Finally, this study did not include data from the COVID‐19 pandemic. Because the COVID‐19 pandemic and the following school closures and social distancing have changed adolescents' movement behaviors substantially, it is important to track the secular trends of the movement guideline adherence among adolescents in the post‐pandemic era to develop interventions to promote healthy behaviors in a timely manner.

## 6 PERSPECTIVE

Adherence to the 24‐h movement guidelines is low and has continually decreased among US adolescents over the past decade. Differences in age, sex and race/ethnicity were all shown to have an impact on whether adolescents meet the public health recommendations for MVPA, recreational ST and sleep concurrently. By quantifying dynamic changes and emerging trends in adolescent 24‐h movement guideline adherence, these findings have the potential to inform where intervention efforts may have the greatest impact for enhancing health outcomes during adolescence.

## AUTHOR CONTRIBUTIONS

**Sitong Chen**: Conceptualization, Methodology, Formal analysis, Writing—original draft, Writing—review & editing; **Denver Brown**: Conceptualization, Writing—review & editing; **Kate Parker**: Conceptualization, Writing—review & editing; **Eun‐Young Lee**: Conceptualization, Writing—review & editing.

## FUNDING INFORMATION

This study receives no external funding.

## CONFLICT OF INTEREST STATEMENT

The authors declared no conflict of interest.

## Supporting information


Table S2.

Table S3.

Table S4.

## Data Availability

Data of this study can be accessed at: https://www.cdc.gov/healthyyouth/data/yrbs/index.htm.

## References

[1] Chaput JP , Carson V , Gray CE , Tremblay MS . Importance of all movement behaviors in a 24 hour period for overall health. Int J Environ Res Public Health. 2014;11:12575‐12581. 25485978 10.3390/ijerph111212575 PMC4276632

[2] Rollo S , Antsygina O , Tremblay MS . The whole day matters: understanding 24‐hour movement guideline adherence and relationships with health indicators across the lifespan. J Sport Health Sci. 2020;9:493‐510. 32711156 10.1016/j.jshs.2020.07.004 PMC7749249

[3] Tremblay MS , Carson V , Chaput JP . Introduction to the canadian 24‐hour movement guidelines for children and youth: an integration of physical activity, sedentary behaviour, and sleep. Appl Physiol Nutr Metab. 2016;41:iii‐iv. 10.1139/apnm-2016-0203 27306430

[4] Pedisic Z , Dumuid D , Olds T . Integrating sleep, sedentary behaviour, and physical activity research in the emerging field of time‐use epidemiology: definitions, concepts, statistical methods, theoretical framework, and future directions. Kinesiology. 2017;49:252‐269.

[5] Tremblay MS , Carson V , Chaput JP , et al. Canadian 24‐hour movement guidelines for children and youth: an integration of physical activity, sedentary behaviour, and sleep. Appl Physiol Nutr Metab. 2016;41:S311‐S327. 27306437 10.1139/apnm-2016-0151

[6] Ross R , Tremblay M . Introduction to the canadian 24‐hour movement guidelines for adults aged 18–64 years and adults aged 65 years or older: an integration of physical activity, sedentary behaviour, and sleep. Appl Physiol Nutr Metab. 2020;45:v‐xi. 33054330 10.1139/apnm-2020-0843

[7] Okely AD , Ghersi D , Loughran SP , et al. A collaborative approach to adopting/adapting guidelines. The australian 24‐hour movement guidelines for children (5‐12 years) and young people (13‐17 years): an integration of physical activity, sedentary behaviour, and sleep. Int J Behav Nutr Phys Act. 2022;19:2. 34991606 10.1186/s12966-021-01236-2 PMC8734238

[8] Knell G , Durand CP , Kohl HW 3rd , Wu IHC , Pettee GK . Prevalence and likelihood of meeting sleep, physical activity, and screen‐time guidelines among us youth. JAMA Pediatr. 2019;173:387‐389. 30715096 10.1001/jamapediatrics.2018.4847 PMC6450269

[9] Janssen I , Roberts KC , Thompson W . Adherence to the 24‐hour movement guidelines among 10‐ to 17‐year‐old canadians. Health Promot Chronic Dis Prev Can. 2017;37:369‐375. 29119774 10.24095/hpcdp.37.11.01 PMC5695900

[10] Chen S‐T , Liu Y , Tremblay MS , et al. Meeting 24‐h movement guidelines: prevalence, correlates, and the relationships with overweight and obesity among chinese children and adolescents. J Sport Health Sci. 2021;10:349‐359. 32679341 10.1016/j.jshs.2020.07.002 PMC8167320

[11] López‐Gil JF , Roman‐Viñas B , Aznar S , Tremblay MS . Meeting 24‐h movement guidelines: prevalence, correlates, and associations with socioemotional behavior in spanish minors. Scand J Med Sci Sports. 2022;32:881‐891. 35090196 10.1111/sms.14132 PMC9303223

[12] Tapia‐Serrano MA , Sevil‐Serrano J , Sánchez‐Miguel PA , López‐Gil JF , Tremblay MS , García‐Hermoso A . Prevalence of meeting 24‐hour movement guidelines from pre‐school to adolescence: a systematic review and meta‐analysis including 387,437 participants and 23 countries. J Sport Health Sci. 2022;11:427‐437. 35066216 10.1016/j.jshs.2022.01.005 PMC9338333

[13] Sampasa‐Kanyinga H , Colman I , Goldfield GS , et al. Combinations of physical activity, sedentary time, and sleep duration and their associations with depressive symptoms and other mental health problems in children and adolescents: a systematic review. Int J Behav Nutr Phys Act. 2020;17:72. 32503638 10.1186/s12966-020-00976-x PMC7273653

[14] Yang Y , Yuan S , Liu Q , et al. Meeting 24‐hour movement and dietary guidelines: prevalence, correlates and association with weight status among children and adolescents: a national cross‐sectional study in China. Nutrients. 2022;14:2822. 35889779 10.3390/nu14142822 PMC9317649

[15] Lee EY , Khan A , Uddin R , Lim E , George L . Six‐year trends and intersectional correlates of meeting 24‐hour movement guidelines among south korean adolescents: Korea Youth Risk Behavior surveys, 2013–2018. J Sport Health Sci. 2023;12:255‐265. 33188965 10.1016/j.jshs.2020.11.001 PMC10105012

[16] Brown DMY , Ronen GM . Associations between 24‐hour movement guideline adherence and mental health disorders among young people with active and inactive epilepsy. Epilepsy Behav. 2021;125:108386. 34781060 10.1016/j.yebeh.2021.108386

[17] Gustavson K , von Soest T , Karevold E , Røysamb E . Attrition and generalizability in longitudinal studies: findings from a 15‐year population‐based study and a monte carlo simulation study. BMC Public Health. 2012;12:918. 23107281 10.1186/1471-2458-12-918 PMC3503744

[18] Mann CJ . Observational research methods. Research design ii: cohort, cross sectional, and case‐control studies. Emerg Med J. 2003;20:54‐60. 12533370 10.1136/emj.20.1.54 PMC1726024

[19] Fernandes HM . Physical activity levels in portuguese adolescents: a 10‐year trend analysis (2006‐2016). J Sci Med Sport. 2018;21:185‐189. 28595866 10.1016/j.jsams.2017.05.015

[20] Sigmund E , Sigmundová D , Badura P , Kalman M , Hamrik Z , Pavelka J . Temporal trends in overweight and obesity, physical activity and screen time among czech adolescents from 2002 to 2014: a national health behaviour in school‐aged children study. Int J Environ Res Public Health. 2015;12:11848‐11868. 26393638 10.3390/ijerph120911848 PMC4586711

[21] Bucksch J , Inchley J , Hamrik Z , Finne E , Kolip P . Trends in television time, non‐gaming pc use and moderate‐to‐vigorous physical activity among german adolescents 2002‐2010. BMC Public Health. 2014;14:351. 24725269 10.1186/1471-2458-14-351 PMC3990022

[22] Bucksch J , Sigmundova D , Hamrik Z , et al. International trends in adolescent screen‐time behaviors from 2002 to 2010. J Adolesc Health. 2016;58:417‐425. 26827267 10.1016/j.jadohealth.2015.11.014

[23] Williams JA , Zimmerman FJ , Bell JF . Norms and trends of sleep time among us children and adolescents. JAMA Pediatr. 2013;167:55‐60. 23403646 10.1001/jamapediatrics.2013.423 PMC5814130

[24] Sawyer SM , Afifi RA , Bearinger LH , et al. Adolescence: a foundation for future health. Lancet. 2012;379:1630‐1640. 22538178 10.1016/S0140-6736(12)60072-5

[25] Booth VM , Rowlands AV , Dollman J . Physical activity temporal trends among children and adolescents. J Sci Med Sport. 2015;18:418‐425. 25041963 10.1016/j.jsams.2014.06.002

[26] Brener ND , Kann L , Shanklin S , et al. Methodology of the Youth Risk Behavior surveillance system—2013. MMWR Recomm Rep. 2013;62:1‐20. 23446553

[27] Brener ND , Collins JL , Kann L , Warren CW , Williams BI . Reliability of the youth risk behavior survey questionnaire. Am J Epidemiol. 1995;141:575‐580. 7900725 10.1093/oxfordjournals.aje.a117473

[28] White IR , Royston P , Wood AM . Multiple imputation using chained equations: issues and guidance for practice. Stat Med. 2011;30:377‐399. 21225900 10.1002/sim.4067

[29] Bang F , Roberts KC , Chaput JP , Goldfield GS , Prince SA . Physical activity, screen time and sleep duration: combined associations with psychosocial health among canadian children and youth. Health Rep. 2020;31:9‐16. 32644766 10.25318/82-003-x202000500002-eng

[30] Roberts KC , Yao X , Carson V , Chaput JP , Janssen I , Tremblay MS . Meeting the canadian 24‐hour movement guidelines for children and youth. Health Rep. 2017;28:3‐7. 29044440

[31] Kuzik N , Cameron C , Carson V , et al. The 2022 participaction report card on physical activity for children and youth: focus on the covid‐19 pandemic impact and equity‐deserving groups. Front Public Health. 2023;11:1172168. 37304090 10.3389/fpubh.2023.1172168 PMC10250634

[32] World Health O . Who Guidelines on Physical Activity and Sedentary Behaviour. World Health Organization; 2020.

[33] Scully M , Gascoyne C , Wakefield M , Morley B . Prevalence and trends in australian adolescents' adherence to 24‐hour movement guidelines: findings from a repeated national cross‐sectional survey. BMC Public Health. 2022;22:105. 35033054 10.1186/s12889-021-12387-z PMC8760722

[34] Felez‐Nobrega M , Raine LB , Haro JM , Wijndaele K , Koyanagi A . Temporal trends in leisure‐time sedentary behavior among adolescents aged 12‐15 years from 26 countries in asia, africa, and the americas. Int J Behav Nutr Phys Act. 2020;17:102. 32787874 10.1186/s12966-020-01010-w PMC7424676

[35] Guthold R , Stevens GA , Riley LM , Bull FC . Global trends in insufficient physical activity among adolescents: a pooled analysis of 298 population‐based surveys with 1·6 million participants. Lancet Child Adolesc Health. 2020;4:23‐35. 31761562 10.1016/S2352-4642(19)30323-2 PMC6919336

[36] Matricciani L , Olds T , Petkov J . In search of lost sleep: secular trends in the sleep time of school‐aged children and adolescents. Sleep Med Rev. 2012;16:203‐211. 21612957 10.1016/j.smrv.2011.03.005

[37] Brown DMY , Kwan MY , Arbour‐Nicitopoulos KP , Cairney J . Identifying patterns of movement behaviours in relation to depressive symptoms during adolescence: a latent profile analysis approach. Prev Med. 2021;143:106352. 33259826 10.1016/j.ypmed.2020.106352

[38] Bauman AE , Reis RS , Sallis JF , Wells JC , Loos RJ , Martin BW . Correlates of physical activity: why are some people physically active and others not? Lancet. 2012;380:258‐271. 22818938 10.1016/S0140-6736(12)60735-1

[39] Butcher K , Sallis JF , Mayer JA , Woodruff S . Correlates of physical activity guideline compliance for adolescents in 100 U.S. Cities J Adolesc Health. 2008;42:360‐368. 18346661 10.1016/j.jadohealth.2007.09.025 PMC2293305

[40] Cheng TL , Goodman E . Race, ethnicity, and socioeconomic status in research on child health. Pediatrics. 2015;135:e225‐e237. 25548336 10.1542/peds.2014-3109 PMC9923597

[41] Lee EY , Airton L , Lim H , Jung E . An urgent need for quantitative intersectionality in physical activity and health research. J Phys Act Health. 2023;20:97‐99. 36634309 10.1123/jpah.2022-0639

[42] Mohammed Abdulaziz F , Kathryn NP , Ashley JA , et al. Timing of the decline in physical activity in childhood and adolescence: Gateshead millennium cohort study. Br J Sports Med. 2018;52:1002‐1006. 28288966 10.1136/bjsports-2016-096933 PMC6204977

