# Supplementary material for: Trends in adherence to the 24‐h movement guidelines among US adolescents from 2011 to 2019: Evidence from repeated cross‐sectional cycles of the Youth Risk Behavior Surveillance System
Source: Scand J Med Sci Sports. 2024 Mar 27;34(4):e14609. doi: 10.1111/sms.14609 (PMC12810440; doi:10.1111/sms.14609)
Supplement: Supplementary file 1 — Table S2. [file SMS-34-e14609-s003.docx]

**Table 2. Sample characteristics by each survey year (2011 – 2019)**

|  |  | **2011** | |  | **Weighted** | | |  | **2013** | |  | **Weighted** | | |  | **2015** | |  | **Weighted** | | |  | **2017** | |  | **Weighted** | | |  | **2019** | |  | **Weighted** | | |
| --- | --- | --- | --- | --- | --- | --- | --- | --- | --- | --- | --- | --- | --- | --- | --- | --- | --- | --- | --- | --- | --- | --- | --- | --- | --- | --- | --- | --- | --- | --- | --- | --- | --- | --- | --- |
|  |  | **n** | **%** |  | **%** | **95% CI** | |  | **n** | **%** |  | **%** | **95% CI** | |  | **n** | **%** |  | **%** | **95% CI** | |  | **n** | **%** |  | **%** | **95% CI** | |  | **n** | **%** |  | **%** | **95%CI** | |
| **Total** |  | 13075 | 100.0 |  | / | | |  | 11219 | 100.0 |  | / | | |  | 13433 | 100.0 |  | / | | |  | 12888 | 100.0 |  | / | | |  | 11974 | 100.0 |  | / | | |
| **Sex** |  |  |  |  |  |  |  |  |  |  |  |  |  |  |  |  |  |  |  |  |  |  |  |  |  |  |  |  |  |  |  |  |  |  |  |
|  | Female | 6650 | 50.9 |  | 49.1 | 47.1 | 51.1 |  | 5569 | 49.6 |  | 50.7 | 49.3 | 52.0 |  | 6819 | 50.8 |  | 49.6 | 46.7 | 52.5 |  | 6690 | 51.9 |  | 51.3 | 48.8 | 53.8 |  | 6162 | 51.5 |  | 49.8 | 48.4 | 51.3 |
|  | Male | 6425 | 49.1 |  | 50.9 | 48.9 | 52.9 |  | 5650 | 50.4 |  | 49.3 | 48.0 | 50.7 |  | 6614 | 49.2 |  | 50.4 | 47.5 | 53.3 |  | 6198 | 48.1 |  | 48.7 | 46.2 | 51.2 |  | 5812 | 48.5 |  | 50.2 | 48.7 | 51.6 |
| **Age** |  |  |  |  |  |  |  |  |  |  |  |  |  |  |  |  |  |  |  |  |  |  |  |  |  |  |  |  |  |  |  |  |  |  |  |
|  | 14 years | 1561 | 11.9 |  | 13.5 | 11.9 | 15.2 |  | 1368 | 12.2 |  | 11.9 | 10.6 | 13.2 |  | 1684 | 12.5 |  | 11.8 | 10.3 | 13.4 |  | 1922 | 14.9 |  | 13.4 | 12.2 | 14.7 |  | 1699 | 14.2 |  | 13.7 | 12.6 | 15.0 |
|  | 15 years | 3470 | 26.5 |  | 28.5 | 27.0 | 30.1 |  | 3098 | 27.6 |  | 28.5 | 27.2 | 29.8 |  | 3817 | 28.4 |  | 30.6 | 29.0 | 32.2 |  | 3586 | 27.8 |  | 28.8 | 27.6 | 30.0 |  | 3473 | 29.0 |  | 28.6 | 27.3 | 30.0 |
|  | 16 years | 4123 | 31.5 |  | 30.5 | 29.4 | 31.7 |  | 3280 | 29.2 |  | 30.5 | 29.0 | 32.1 |  | 4099 | 30.5 |  | 29.9 | 28.2 | 31.6 |  | 3769 | 29.2 |  | 29.9 | 28.6 | 31.2 |  | 3700 | 30.9 |  | 30.2 | 29.1 | 31.4 |
|  | 17 years | 3921 | 30.0 |  | 27.5 | 25.9 | 29.2 |  | 3473 | 31.0 |  | 29.1 | 27.8 | 30.4 |  | 3833 | 28.5 |  | 27.8 | 26.6 | 29.0 |  | 3611 | 28.0 |  | 27.9 | 26.6 | 29.3 |  | 3102 | 25.9 |  | 27.4 | 26.1 | 28.6 |
| **Race** |  |  |  |  |  |  |  |  |  |  |  |  |  |  |  |  |  |  |  |  |  |  |  |  |  |  |  |  |  |  |  |  |  |  |  |
|  | White | 5409 | 41.4 |  | 56.3 | 49.0 | 63.4 |  | 4605 | 41.0 |  | 55.0 | 47.0 | 62.7 |  | 6104 | 45.4 |  | 54.0 | 46.3 | 61.5 |  | 5691 | 44.2 |  | 53.5 | 46.9 | 60.0 |  | 6024 | 50.3 |  | 50.6 | 43.6 | 57.6 |
|  | Black or African American | 2331 | 17.8 |  | 14.1 | 10.8 | 18.3 |  | 2458 | 21.9 |  | 14.3 | 10.1 | 19.8 |  | 1460 | 10.9 |  | 13.8 | 10.4 | 18.0 |  | 2472 | 19.2 |  | 13.5 | 10.7 | 17.0 |  | 1837 | 15.3 |  | 12.4 | 9.2 | 16.4 |
|  | Hispanic/Latino | 3969 | 30.4 |  | 20.4 | 15.5 | 26.4 |  | 2905 | 25.9 |  | 21.5 | 16.8 | 27.1 |  | 4425 | 32.9 |  | 22.5 | 16.9 | 29.3 |  | 3175 | 24.6 |  | 22.8 | 17.6 | 28.9 |  | 2750 | 23.0 |  | 26.4 | 20.7 | 33.0 |
|  | All other races | 1366 | 10.4 |  | 9.2 | 7.3 | 11.4 |  | 1251 | 11.2 |  | 9.2 | 7.5 | 11.3 |  | 1444 | 10.7 |  | 9.7 | 7.7 | 12.2 |  | 1550 | 12.0 |  | 10.2 | 8.7 | 12.0 |  | 1363 | 11.4 |  | 10.6 | 8.0 | 14.0 |
| **Physical activity guidelines** |  |  |  |  |  |  |  |  |  |  |  |  |  |  |  |  |  |  |  |  |  |  |  |  |  |  |  |  |  |  |  |  |  |  |  |
|  | Not met | 9506 | 72.7 |  | 71.0 | 69.4 | 72.7 |  | 9466 | 72.4 |  | 72.7 | 71.1 | 74.4 |  | 9715 | 74.3 |  | 72.6 | 70.7 | 74.4 |  | 9859 | 75.4 |  | 73.5 | 71.2 | 75.7 |  | 10081 | 77.1 |  | 76.2 | 74.8 | 77.5 |
|  | Met | 3569 | 27.3 |  | 29.0 | 27.3 | 30.6 |  | 3609 | 27.6 |  | 27.3 | 25.6 | 28.9 |  | 3360 | 25.7 |  | 27.4 | 25.6 | 29.3 |  | 3216 | 24.6 |  | 26.5 | 24.3 | 28.8 |  | 2994 | 22.9 |  | 23.8 | 22.5 | 25.2 |
| **Screen time guidelines** |  |  |  |  |  |  |  |  |  |  |  |  |  |  |  |  |  |  |  |  |  |  |  |  |  |  |  |  |  |  |  |  |  |  |  |
|  | Not met | 9702 | 74.2 |  | 73.3 | 71.9 | 74.8 |  | 10029 | 76.7 |  | 74.6 | 72.2 | 76.9 |  | 9349 | 71.5 |  | 70.6 | 68.6 | 72.6 |  | 8996 | 68.8 |  | 68.6 | 66.4 | 70.9 |  | 9061 | 69.3 |  | 69.9 | 68.3 | 71.5 |
|  | Met | 3373 | 25.8 |  | 26.7 | 25.2 | 28.1 |  | 3046 | 23.3 |  | 25.4 | 23.1 | 27.8 |  | 3726 | 28.5 |  | 29.4 | 27.4 | 31.4 |  | 4079 | 31.2 |  | 31.4 | 29.1 | 33.6 |  | 4014 | 30.7 |  | 30.1 | 28.5 | 31.7 |
| **Sleep guidelines** |  |  |  |  |  |  |  |  |  |  |  |  |  |  |  |  |  |  |  |  |  |  |  |  |  |  |  |  |  |  |  |  |  |  |  |
|  | Not met | 8996 | 68.8 |  | 68.1 | 66.7 | 69.6 |  | 8930 | 68.3 |  | 67.2 | 65.6 | 68.9 |  | 9309 | 71.2 |  | 71.9 | 69.6 | 74.1 |  | 9610 | 73.5 |  | 73.4 | 71.8 | 74.9 |  | 10042 | 76.8 |  | 77.1 | 75.5 | 78.6 |
|  | Met | 4079 | 31.2 |  | 31.9 | 30.4 | 33.3 |  | 4145 | 31.7 |  | 32.8 | 31.1 | 34.4 |  | 3766 | 28.8 |  | 28.1 | 25.9 | 30.4 |  | 3465 | 26.5 |  | 26.6 | 25.1 | 28.2 |  | 3033 | 23.2 |  | 22.9 | 21.4 | 24.5 |
| **24-hour movement guidelines** |  |  |  |  |  |  |  |  |  |  |  |  |  |  |  |  |  |  |  |  |  |  |  |  |  |  |  |  |  |  |  |  |  |  |  |
|  | Not meet | 12651 | 96.8 |  | 96.4 | 95.9 | 96.9 |  | 10878 | 97.0 |  | 96.6 | 95.9 | 97.1 |  | 12976 | 96.6 |  | 96.6 | 96.0 | 97.2 |  | 12473 | 96.8 |  | 97.4 | 97.0 | 97.7 |  | 11662 | 97.4 |  | 97.4 | 97.0 | 97.7 |
|  | Meet | 424 | 3.2 |  | 3.6 | 3.1 | 4.1 |  | 341 | 3.0 |  | 3.4 | 2.9 | 4.1 |  | 457 | 3.4 |  | 3.4 | 2.8 | 4.0 |  | 415 | 3.2 |  | 2.6 | 2.3 | 3.0 |  | 312 | 2.6 |  | 2.6 | 2.3 | 3.0 |

CI: confidence interval.
