# Supplementary material for: Trends in adherence to the 24‐h movement guidelines among US adolescents from 2011 to 2019: Evidence from repeated cross‐sectional cycles of the Youth Risk Behavior Surveillance System
Source: Scand J Med Sci Sports. 2024 Mar 27;34(4):e14609. doi: 10.1111/sms.14609 (PMC12810440; doi:10.1111/sms.14609)
Supplement: Supplementary file 2 — Table S3. [file SMS-34-e14609-s002.docx]

**Table 3. Demographic correlates of meeting the 24-hour movement guidelines in the overall sample and by different survey years.**

|  |  | **Total ⃰**  **(n = 62589)** | | |  | **2011**  **(n = 13075)** | | |  | **2013**  **(n = 11219)** | | |  | **2015**  **(n = 13433)** | | |  | **2017**  **(n = 12888)** | | |  | **2019**  **(n = 11974)** | | |
| --- | --- | --- | --- | --- | --- | --- | --- | --- | --- | --- | --- | --- | --- | --- | --- | --- | --- | --- | --- | --- | --- | --- | --- | --- |
|  |  | **OR** | **95%CI** | |  | **OR** | **95%CI** | |  | **OR** | **95%CI** | |  | **OR** | **95%CI** | |  | **OR** | **95%CI** | |  | **OR** | **95%CI** | |
| Sex ^a^ |  |  |  |  |  |  |  |  |  |  |  |  |  |  |  |  |  |  |  |  |  |  |  |  |
|  | Male | 2.16 | 1.89 | 2.46 |  | 1.81 | 1.43 | 2.29 |  | 2.22 | 1.63 | 3.03 |  | 2.43 | 1.78 | 3.31 |  | 2.19 | 1.59 | 3.01 |  | 2.25 | 1.64 | 3.08 |
|  | Female | Ref | | |  | Ref | | |  | Ref | | |  | Ref | | |  | Ref | | |  | Ref | | |
|  |  |  |  |  |  |  |  |  |  |  |  |  |  |  |  |  |  |  |  |  |  |  |  |  |
| Age group ^b^ |  |  |  |  |  |  |  |  |  |  |  |  |  |  |  |  |  |  |  |  |  |  |  |  |
|  | 14 years | 1.85 | 1.51 | 2.26 |  | 1.86 | 1.29 | 2.67 |  | 1.72 | 1.01 | 2.94 |  | 1.47 | 1.05 | 2.05 |  | 1.99 | 1.21 | 3.27 |  | 2.31 | 1.50 | 3.56 |
|  | 15 years | 1.67 | 1.41 | 1.98 |  | 1.76 | 1.11 | 2.80 |  | 1.06 | 0.70 | 1.61 |  | 2.17 | 1.57 | 3.00 |  | 1.73 | 1.24 | 2.41 |  | 1.81 | 1.28 | 2.55 |
|  | 16 years | 1.36 | 1.15 | 1.60 |  | 1.11 | 0.72 | 1.69 |  | 1.15 | 0.80 | 1.66 |  | 1.95 | 1.40 | 2.70 |  | 1.29 | 0.93 | 1.81 |  | 1.53 | 1.00 | 2.33 |
|  | 17 years | Ref | | |  | Ref | | |  | Ref | | |  | Ref | | |  | Ref | | |  | Ref | | |
|  |  |  |  |  |  |  |  |  |  |  |  |  |  |  |  |  |  |  |  |  |  |  |  |  |
| Race/ethnicity ^c^ |  |  |  |  |  |  |  |  |  |  |  |  |  |  |  |  |  |  |  |  |  |  |  |  |
|  | White | 2.48 | 2.00 | 3.09 |  | 1.78 | 1.27 | 2.48 |  | 3.05 | 1.73 | 5.40 |  | 2.37 | 1.32 | 4.27 |  | 3.12 | 1.76 | 5.52 |  | 2.98 | 1.55 | 5.70 |
|  | Hispanic/Latino | 1.86 | 1.47 | 2.36 |  | 1.24 | 0.83 | 1.88 |  | 2.71 | 1.63 | 4.49 |  | 1.73 | 0.97 | 3.10 |  | 2.26 | 1.23 | 4.15 |  | 2.16 | 1.07 | 4.37 |
|  | All other races** | 1.57 | 1.17 | 2.11 |  | 1.44 | 0.84 | 2.49 |  | 1.27 | 0.62 | 2.62 |  | 1.47 | 0.78 | 2.78 |  | 2.36 | 1.21 | 4.58 |  | 1.49 | 0.64 | 3.49 |
|  | Black or African American | Ref | | |  | Ref | | |  | Ref | | |  | Ref | | |  | Ref | | |  | Ref | | |

OR: odds ratio; CI: confidence interval; Ref: reference group.

⃰ Model for the overall sample controlled for survey year.

**All other races included American Indian/Alaska Native, Asian, Native Hawaiian/other Pacific Islanders, and Multiple - Non-Hispanic.

^a^ Results were adjusted for age, and race/ethnicity.

^b^ Results were adjusted for sex, and race/ethnicity.

^c^ Results were adjusted for sex, and age.
