# Supplementary material for: Trends in adherence to the 24‐h movement guidelines among US adolescents from 2011 to 2019: Evidence from repeated cross‐sectional cycles of the Youth Risk Behavior Surveillance System
Source: Scand J Med Sci Sports. 2024 Mar 27;34(4):e14609. doi: 10.1111/sms.14609 (PMC12810440; doi:10.1111/sms.14609)
Supplement: Supplementary file 3 — Table S4. [file SMS-34-e14609-s001.docx]

**Table 4. Prevalence of the adherence to the physical activity, screen time and sleep guidelines (separately) in the overall sample and sample by sex, age and race/ethnicity**

|  |  | **2011 year** | | |  | **2013 year** | | |  | **2015 year** | | |  | **2017 year** | | |  | **2019 year** | | |  |
| --- | --- | --- | --- | --- | --- | --- | --- | --- | --- | --- | --- | --- | --- | --- | --- | --- | --- | --- | --- | --- | --- |
| **Specific guidelines meet** | **By group** | **%** | **95%CI** | |  | **%** | **95%CI** | |  | **%** | **95%CI** | |  | **%** | **95%CI** | |  | **%** | **95%CI** | |  |
| **Physical activity guidelines** |  |  |  |  |  |  |  |  |  |  |  |  |  |  |  |  |  |  |  |  |  |
|  | Overall ^a^ | 29.0 | 27.3 | 30.6 |  | 27.3 | 25.6 | 28.9 |  | 27.4 | 25.6 | 29.3 |  | 26.5 | 24.3 | 28.8 |  | 23.8 | 22.5 | 25.2 |  |
|  |  |  |  |  |  |  |  |  |  |  |  |  |  |  |  |  |  |  |  |  |  |
|  | Sex ^b^ |  |  |  |  |  |  |  |  |  |  |  |  |  |  |  |  |  |  |  |  |
|  | Male | 38.6 | 36.5 | 40.7 |  | 36.9 | 34.9 | 38.9 |  | 36.4 | 33.8 | 39.0 |  | 36.2 | 34.0 | 38.5 |  | 31.5 | 29.5 | 33.6 |  |
|  | Female | 19.0 | 17.2 | 20.8 |  | 17.9 | 16.2 | 19.6 |  | 18.3 | 16.8 | 19.8 |  | 17.4 | 15.3 | 19.4 |  | 16.0 | 14.6 | 17.4 |  |
|  |  |  |  |  |  |  |  |  |  |  |  |  |  |  |  |  |  |  |  |  |  |
|  | Age group ^c^ |  |  |  |  |  |  |  |  |  |  |  |  |  |  |  |  |  |  |  |  |
|  | 14 year | 31.6 | 28.1 | 35.0 |  | 30.2 | 26.6 | 33.8 |  | 25.7 | 23.2 | 28.3 |  | 26.8 | 23.2 | 30.4 |  | 27.0 | 23.6 | 30.5 |  |
|  | 15 year | 29.9 | 27.4 | 32.5 |  | 28.4 | 25.9 | 30.9 |  | 30.7 | 27.6 | 33.7 |  | 28.7 | 26.0 | 31.3 |  | 25.8 | 23.5 | 28.1 |  |
|  | 16 year | 29.1 | 26.9 | 31.2 |  | 27.6 | 25.6 | 29.5 |  | 26.6 | 24.5 | 28.8 |  | 25.4 | 22.4 | 28.3 |  | 22.3 | 20.1 | 24.5 |  |
|  | 17 year | 26.6 | 24.7 | 28.6 |  | 24.7 | 22.4 | 27.0 |  | 25.4 | 22.9 | 27.9 |  | 25.5 | 23.1 | 28.0 |  | 21.8 | 19.5 | 24.1 |  |
|  |  |  |  |  |  |  |  |  |  |  |  |  |  |  |  |  |  |  |  |  |  |
|  | Race/ethnicity ^d^ |  |  |  |  |  |  |  |  |  |  |  |  |  |  |  |  |  |  |  |  |
|  | White | 30.5 | 28.2 | 32.8 |  | 28.1 | 25.9 | 30.4 |  | 29.3 | 26.7 | 31.9 |  | 27.5 | 24.6 | 30.4 |  | 26.6 | 25.1 | 28.1 |  |
|  | Black or African American | 26.6 | 24.3 | 28.9 |  | 26.7 | 24.0 | 29.3 |  | 25.3 | 24.8 | 22.0 |  | 21.3 | 20.9 | 18.5 |  | 29.3 | 28.6 | 25.5 |  |
|  | Hispanic/Latino | 27.2 | 25.3 | 29.1 |  | 26.0 | 23.4 | 28.6 |  | 24.6 | 22.0 | 27.2 |  | 26.3 | 23.6 | 28.9 |  | 21.1 | 18.5 | 23.7 |  |
|  | All other races | 27.3 | 24.2 | 30.4 |  | 26.2 | 22.9 | 29.6 |  | 26.5 | 22.7 | 30.4 |  | 24.8 | 21.2 | 28.4 |  | 19.4 | 16.8 | 21.9 |  |
|  |  |  |  |  |  |  |  |  |  |  |  |  |  |  |  |  |  |  |  |  |  |
| **Screen time guidelines** |  |  |  |  |  |  |  |  |  |  |  |  |  |  |  |  |  |  |  |  |  |
|  | Overall ^a^ |  |  |  |  |  |  |  |  |  |  |  |  |  |  |  |  |  |  |  |  |
|  |  | 26.7 | 25.2 | 28.1 |  | 25.4 | 23.1 | 27.8 |  | 29.4 | 27.4 | 31.4 |  | 31.4 | 29.1 | 33.6 |  | 30.1 | 28.5 | 31.7 |  |
|  | Sex ^b^ |  |  |  |  |  |  |  |  |  |  |  |  |  |  |  |  |  |  |  |  |
|  | Male | 21.5 | 21.5 | 24.2 |  | 20.9 | 20.9 | 25.7 |  | 25.5 | 25.5 | 31.1 |  | 27.5 | 27.5 | 31.4 |  | 26.3 | 26.3 | 30.5 |  |
|  | Female | 30.6 | 28.4 | 32.9 |  | 27.5 | 24.4 | 30.6 |  | 30.4 | 28.4 | 32.5 |  | 33.2 | 29.3 | 37.1 |  | 31.8 | 30.0 | 33.5 |  |
|  |  |  |  |  |  |  |  |  |  |  |  |  |  |  |  |  |  |  |  |  |  |
|  | Age group ^c^ |  |  |  |  |  |  |  |  |  |  |  |  |  |  |  |  |  |  |  |  |
|  | 14 year | 26.9 | 23.7 | 30.2 |  | 23.1 | 18.9 | 27.2 |  | 25.0 | 21.4 | 28.6 |  | 28.5 | 24.6 | 32.4 |  | 28.3 | 25.1 | 31.5 |  |
|  | 15 year | 25.6 | 23.2 | 28.0 |  | 23.0 | 20.1 | 26.0 |  | 27.9 | 25.0 | 30.8 |  | 29.4 | 26.6 | 32.1 |  | 29.4 | 26.9 | 31.8 |  |
|  | 16 year | 26.3 | 24.7 | 27.9 |  | 26.8 | 23.4 | 30.1 |  | 30.5 | 28.3 | 32.6 |  | 30.6 | 27.6 | 33.5 |  | 30.5 | 27.8 | 33.2 |  |
|  | 17 year | 28.1 | 25.9 | 30.3 |  | 27.3 | 25.1 | 29.4 |  | 31.7 | 28.5 | 34.8 |  | 35.7 | 33.5 | 37.9 |  | 31.2 | 28.9 | 33.5 |  |
|  |  |  |  |  |  |  |  |  |  |  |  |  |  |  |  |  |  |  |  |  |  |
|  | Race/ethnicity ^d^ |  |  |  |  |  |  |  |  |  |  |  |  |  |  |  |  |  |  |  |  |
|  | White | 29.3 | 27.4 | 31.1 |  | 29.0 | 25.0 | 32.9 |  | 31.2 | 28.3 | 34.1 |  | 32.9 | 29.1 | 36.6 |  | 30.4 | 28.5 | 32.3 |  |
|  | Black or African American | 18.6 | 15.2 | 22.1 |  | 17.5 | 14.7 | 20.3 |  | 26.0 | 22.7 | 29.3 |  | 24.8 | 22.1 | 27.6 |  | 29.0 | 25.1 | 32.9 |  |
|  | Hispanic/Latino | 25.4 | 23.1 | 27.7 |  | 22.7 | 20.7 | 24.8 |  | 27.4 | 25.3 | 29.5 |  | 31.7 | 29.4 | 34.0 |  | 29.7 | 27.3 | 32.1 |  |
|  | All other races | 25.8 | 22.9 | 28.7 |  | 22.8 | 19.1 | 26.5 |  | 28.2 | 22.7 | 33.6 |  | 31.4 | 27.0 | 35.7 |  | 30.7 | 26.6 | 34.9 |  |
|  |  |  |  |  |  |  |  |  |  |  |  |  |  |  |  |  |  |  |  |  |  |
| **Sleep guidelines** |  |  |  |  |  |  |  |  |  |  |  |  |  |  |  |  |  |  |  |  |  |
|  | Overall ^a^ |  |  |  |  |  |  |  |  |  |  |  |  |  |  |  |  |  |  |  |  |
|  |  | 31.9 | 30.4 | 33.3 |  | 32.8 | 31.1 | 34.4 |  | 28.1 | 25.9 | 30.4 |  | 26.6 | 25.1 | 28.2 |  | 22.9 | 21.4 | 24.5 |  |
|  | Sex ^b^ |  |  |  |  |  |  |  |  |  |  |  |  |  |  |  |  |  |  |  |  |
|  | Male | 34.3 | 32.3 | 36.2 |  | 36.1 | 33.9 | 38.3 |  | 31.4 | 28.5 | 34.3 |  | 27.9 | 26.0 | 29.9 |  | 25.0 | 23.4 | 26.7 |  |
|  | Female | 29.4 | 27.7 | 31.1 |  | 29.5 | 27.6 | 31.5 |  | 24.8 | 22.5 | 27.1 |  | 25.4 | 23.5 | 27.2 |  | 20.8 | 18.8 | 22.8 |  |
|  |  |  |  |  |  |  |  |  |  |  |  |  |  |  |  |  |  |  |  |  |  |
|  | Age group ^c^ |  |  |  |  |  |  |  |  |  |  |  |  |  |  |  |  |  |  |  |  |
|  | 14 year | 40.3 | 37.1 | 43.6 |  | 41.7 | 37.8 | 45.6 |  | 36.7 | 33.2 | 40.3 |  | 33.5 | 30.6 | 36.4 |  | 30.0 | 26.6 | 33.4 |  |
|  | 15 year | 36.5 | 34.0 | 39.0 |  | 36.4 | 33.9 | 38.8 |  | 30.9 | 27.3 | 34.5 |  | 32.2 | 29.8 | 34.6 |  | 25.7 | 23.1 | 28.2 |  |
|  | 16 year | 29.8 | 27.6 | 32.0 |  | 30.7 | 28.1 | 33.3 |  | 27.1 | 24.3 | 30.0 |  | 24.6 | 22.2 | 27.0 |  | 22.3 | 20.2 | 24.3 |  |
|  | 17 year | 25.2 | 23.0 | 27.3 |  | 27.8 | 25.4 | 30.2 |  | 22.5 | 19.8 | 25.2 |  | 19.6 | 18.1 | 21.1 |  | 17.3 | 15.6 | 18.9 |  |
|  |  |  |  |  |  |  |  |  |  |  |  |  |  |  |  |  |  |  |  |  |  |
|  | Race/ethnicity ^d^ |  |  |  |  |  |  |  |  |  |  |  |  |  |  |  |  |  |  |  |  |
|  | White | 33.2 | 31.3 | 35.1 |  | 33.5 | 31.0 | 36.1 |  | 28.9 | 26.4 | 31.4 |  | 27.8 | 26.0 | 29.7 |  | 24.1 | 22.0 | 26.1 |  |
|  | Black or African American | 29.3 | 26.1 | 32.5 |  | 29.6 | 26.5 | 32.7 |  | 24.4 | 20.5 | 28.3 |  | 25.2 | 22.5 | 28.0 |  | 21.0 | 17.7 | 24.3 |  |
|  | Hispanic/Latino | 31.0 | 28.9 | 33.2 |  | 33.9 | 31.5 | 36.2 |  | 30.6 | 27.1 | 34.1 |  | 27.4 | 24.7 | 30.1 |  | 22.6 | 20.3 | 24.9 |  |
|  | All other races | 29.4 | 25.3 | 33.4 |  | 30.6 | 27.2 | 34.0 |  | 23.6 | 19.7 | 27.4 |  | 20.3 | 17.5 | 23.0 |  | 20.5 | 17.4 | 23.6 |  |

^a^ Results were adjusted for sex, age and race/ethnicity.

^b^ Results were adjusted for age and race/ethnicity.

^c^ Results were adjusted for sex and race/ethnicity.

^d^ Results were adjusted for sex and age.
